# Augmenting progress on the elimination of vertical transmissions of HIV in India: Insights from Spectrum-based HIV burden estimations

**DOI:** 10.1371/journal.pgph.0002270

**Published:** 2023-08-09

**Authors:** Pradeep Kumar, Chinmoyee Das, Udayabhanu Das, Arvind Kumar, Nidhi Priyam, Varsha Ranjan, Damodar Sahu, Sanjay K. Rai, Sheela V. Godbole, Elangovan Arumugam, Lakshmi PVM, Shanta Dutta, H. Sanayaima Devi, Arvind Pandey, Dandu Chandra Sekhar Reddy, Sanjay Mehendale, Shobini Rajan

**Affiliations:** 1 National AIDS Control Organization, Ministry of Health and Family Welfare, New Delhi, India; 2 Indian Council of Medical Research, National Institute of Medical Statistics, New Delhi, India; 3 All India Institute of Medical Sciences, New Delhi, India; 4 Indian Council of Medical Research, National AIDS Research Institute, Pune, India; 5 Indian Council of Medical Research, National Institute of Epidemiology, Chennai, India; 6 Postgraduate Institute of Medical Education and Research, Chandigarh, India; 7 Indian Council of Medical Research, National Institute of Cholera and Enteric Diseases, Kolkata, India; 8 Regional Institute of Medical Sciences, Imphal, India; 9 Indian Council of Medical Research, New Delhi, India; 10 Institute of Medical Sciences, Banaras Hindu University, Varanasi, India; 11 PD Hinduja Hospital and Medical Research Center, Mumbai, India; PLOS: Public Library of Science, UNITED STATES

## Abstract

The government of India has adopted the elimination of vertical transmission of HIV as one of the five high-level goals under phase V of the National AIDS and STD Control Programme (NACP). In this paper, we present the data from HIV estimations 2021 for India and select States detailing the progress as well as the attributable causes for vertical transmissions. The NACP spearheads work on mathematical modelling to estimate HIV burden based on the periodically conducted sentinel surveillance for guiding program implementation and policymaking. Using the results of the latest round of HIV Estimations in 2021, we analysed the mother-to-child transmission (MTCT) during the perinatal and postnatal (breastfeeding) period. In 2021, overall, around 5,000 [3,000–7,800] vertical transmissions were estimated nationally with 58% being perinatal infections and remaining during breastfeeding. MTCT at 6 weeks was around 12.95% [9.45–16.02] with the final transmission rate at 24.25% [18.50–29.50]. Overall, 57% of vertical transmissions were among HIV-positive mothers who did not receive ART during pregnancy or breastfeeding, 19% among mothers who dropped off ART during pregnancy or delivery, and 18% among mothers who were infected during pregnancy or breastfeeding. There were significant variations between States. Depending upon the States, the programme needs to focus on the intervention domains of timely engagement in antenatal care-HIV testing-ART initiation as well as programme retention and adherence support. Equally important would be strengthening the strategic information to generate related evidence for inputting India and State-specific parameters improving the MTCT-related modelled estimates.

## Introduction

United Nations envisages ending the AIDS epidemic as a public health threat by 2030 as one of the targets (Target 3.3) under the third Sustainable Development Goal (SDG) of ensuring healthy lives and promoting well-being for all at all ages [1]. Elimination of vertical transmission of HIV (EVTH), reflected by the global HIV response in its pledge to zero new HIV infections, zero AIDS-related deaths and zero HIV-related discrimination, is integral to the attainment of target 3.3 [2, 3]. The elimination of vertical transmission of HIV has been defined in scientific terms. World Health Organization (WHO) has released specific guidance periodically to measure the progress on EVTH. Attainment of a population case rate of new paediatric HIV infections due to vertical transmission of ≤50 cases per 100 000 live births and mother-to-child-transmission (MTCT) rate of HIV of <2% in non-breastfeeding populations OR <5% in breastfeeding populations are two impact targets for EVTH [4].

There has been significant progress on EVTH. As of November 2021, WHO has validated fourteen countries/territories including Cuba, Thailand, Belarus, Armenia, Anguilla, Montserrat, Cayman Islands, Bermuda, Antigua and Barbuda, St Christopher and Nevis, Malaysia, Maldives, Sri Lanka and Dominica for EVTH [4]. The annual vertical transmissions between 2010 and 2021 have decreased by fifty per cent. Still, with around 160,000 (110,000–230,000) vertical transmissions in 2021, progress on EVTH is far from satisfactory [5].

India, the second largest HIV epidemic with an estimated 2.4 million [1.99–2.90 million] people living with HIV (PLHIV) in 2021, is critical to the global AIDS response [6]. Being committed to the attainment of ending AIDS as a public health threat by 2030, the Government of India has adopted new strategies and targets under phase V of the National AIDS and STD Control Programme (NACP Phase-V) [7]. NACP Phase-V has five top-level goals and 23 output/outcome targets to anchor the national HIV response till 2025–26. EVTH is explicitly stated as Goal 3 of NACP Phase-V.

Efforts to prevent and eliminate vertical transmission of HIV under NACP are not new. Direct interventions for the prevention of vertical transmission of HIV in India were initiated as early as 2002 [8, 9]. The focus gradually shifted to attain elimination of vertical transmission by 2020 as one of the priorities areas under NACP through four-pronged strategies of primary prevention of HIV, prevention of unintended pregnancies, prevention of vertical transmission, and care, support, and treatment of women living with HIV (WLHIV) and her children. As a result of the focus on primary preventions under NACP with new infections declining by more than 80% since the peak, the case rate of vertical pediatric HIV infections declined to 24 per 100,000 live births in 2021. MTCT rate, reflecting the treatment coverages among HIV-positive pregnant and breastfeeding women, declined from ≥ 40% in 2010 to 24% in 2021. Still, NACP has a long way to go to attain the target MTCT rate of <5% for the elimination of paediatric new infections [6, 9].

MTCT rate under NACP of India is calculated using UNAIDS recommended Spectrum Model. This is consistent with global recommendations to estimate the population-level MTCT rate [4]. To better understand the vertical transmission of HIV in India, we present the first disaggregated estimates of the vertical transmission of HIV acquired during pregnancy, delivery and breastfeeding in India. We provide this estimate not only for the national level but also for the high HIV burden States (either PLHIV size of ≥ 50,000 PLHIV or adult HIV prevalence of ≥1%) increasing the granularity of analysis for informing the tailored policy-making and interventions designed to augment the progress on EVTH under NACP in India.

## Methods

### Ethics statement

For the present analysis, we used aggregated deidentified outputs of HIV Estimations 2021 to quantify the vertical transmissions during different phases of motherhood. HIV burden estimations under the NACP of India are undertaken periodically and are the outcome of robust epidemic monitoring techniques implemented through an institutionalised mechanism of Surveillance & Epidemiology informing policy-making and programmatic improvements [10]. The institutions involved in primary data collection through periodic HIV sero-surveillance submit their proposals for the surveillance program using globally recommended methods to their respective ethics committees to seek approval on the informed consent forms. The survey at each site is initiated after the approval of the local ethics committee. The epidemic data thus generated, along with programmatic data, is used for HIV burden estimations by employing the Spectrum model, developed by Avenir Health, UNAIDS and partners [11]. As this study used aggregated de-identified outputs generated through the HIV Estimations 2021 model, ethical approval was not required.

### MTCT estimation under Spectrum

The details of the process and method for the HIV burden estimations through Spectrum, used by 170 countries representing 99% of the global population, have been described elsewhere [12–18]. The model assumptions are reviewed and updated periodically by the multi-disciplinary UNAIDS reference group on Estimates, Modelling and Projections.

In brief, country teams input demographics, programmatic and HIV prevalence data among 15–49 years old in the model. The model transforms prevalence trends into incidence trends based on the inputted data about antiretroviral therapy (ART) coverage and assumptions about CD4 progression and survival on and off ART. The incidence estimates are then distributed by age and sex based on the community-based survey data or the epidemic type and then progressed over time to death depending upon coverage of the ART programme.

WLHIV in the age group of 15–49 years are subjected to fertility rates. The number of WLHIV in each age group is multiplied by total fertility rates and the age distribution of fertility in that age group as given in the formula below [18, 19]. The adjustment is done to account for the impact of HIV infection on fertility [20, 21].

$${\text{BW}}_{\text{t}}=\Sigma \;{\text{W}}_{\text{a},\text{t}}\times {\text{TFR}}_{\text{t}}\times {\text{ASFR}}_{\text{a},\text{t}}$$

Where;

BW_t_ = the number of births occurring among WLHIV in year ‘t’; W_a,t_ = the number of WLHIV of age ‘a’ at time ‘t’; TFR_t_ = the total fertility rate at time ‘t’, and; ASFR_a,t_ = the percentage of life time births that occur to women of age ‘a’ at time ‘t’

The model estimates the perinatal vertical transmission rate as the weighted average of the proportion in each of the 11 prophylaxis/treatment group and the corresponding probability of transmission [20]. The prophylaxis/treatment groups include: No prophylaxis (in three category of CD4 count of <200 cells/ /μl, 200–350 cells/μl and >350 cells/μl), incident infections, single dose Neviripine, Dual ARVs, Option A, Option B, Option B+ (in three category of ART started before current pregnancy, ART started during current pregnancy more than 4 weeks before delivery and ART started during current pregnancy less than 4 weeks before delivery).

$${\text{PTR}}_{\text{t}}=\Sigma {\text{Prophylaxis}}_{\text{c},\text{t}}\times {\text{TR}}_{\text{c}}$$

Where, PTR_t_ = Perinatal transmission rate; Prophylaxis_c,t_ = the proportion of women by prophylaxis/treatment category, and; TR_c_ = probability of transmission of HIV by prophylaxis/treatment category.

The transmission probabiliby in Spectrum model vary by each of the prophylaxis/treatment group (Table 1) and are based on the expert review of available studies [16, 22]. In the model, the risk of vertical transmission is highest when the HIV infections in women occus while pregnant or breastfeeding (incident infections). The probability of vertical transmission with incident infections during pregnancy is estimated at around 18%, and 27% for those occurring during breastfeeding. WLHIV who were on antiretroviral therapy prior to pregnancy have the lowest peripartum and postnatal transmission probabilities; 0.26% for peripartum and 0.02% per month of breastfeeding, respectively.

**Table 1:** Peripartum and postpartum probability of HIV transmission by prophylaxis/treatment regimen (%).

Regimen	Perinatal	Breastfeeding (per month)	
		< 350	> = 350
No prophylaxis			
Existing infections			
CD4 < 200	37	0.89	
CD4 200–350	27	0.81	
CD4 > 350	15		0.51
Incident infections	18.1	26.9	26.9
Single dose nevirapine	7.5	0.99	0.4
WHO 2006 dual ARV regimen	2.2	0.18	0.18
Option A	4.1		0.2
Option B	1.9		0.13
ART			
Started before pregnancy	0.26	0.02	
Started during pregnancy > 4 weeks	1.4	0.11	
Started during pregnancy < 4 weeks	8.2	0.2	

The model estimates the number of HIV+ births by applying the perinatal transmission rate to the number of births among WLHIV [20].

$${\text{B}}_{\text{HIV}+,\text{t}}={\text{BW}}_{\text{t}}\times {\text{PTR}}_{\text{t}}$$

Where; B_HIV+,t_ = the number of HIV+ births; BW_t_ = the number of births occurring among WLHIV in year ‘t’; PTR_t_ = Perinatal transmission rate.

The model estimates the postnatal vertical transmission as product of the number of children born to HIV+ mothers who were not infected perinatally (= BWt- B_HIV+,t_), the proportion of children exposed to transmission through breastfeeding, the monthly probability of transmission through breastfeeding (on and off-ART) and the duration of breastfeeding [20]. Postnatal transmisison is estimated for each month from birth to 36 months.

$$\begin{array}{rr} {\text{BFTR}}_{\text{m},\text{t}} & =\text{No}\_{\text{prophylaxis}}_{\text{m},\text{t}}\times \left[{\text{Prop}}_{\text{LT}350}\times {\text{TR}}_{\text{LT}350}+\left(1-{\text{Prop}}_{\text{LT}350}\right)\times {\text{TR}}_{\text{GT}350}+{\text{PropInc}}_{\text{t}}\times {\text{TR}}_{\text{i}}\right]\\ & +{\text{OptA}}_{\text{m},\text{t}}\times {\text{TR}}_{\text{OptA}}+{\text{OptB}}_{\text{m},\text{t}}\times {\text{TR}}_{\text{OptB}}+{\text{ARTbefore}}_{\text{t}}\times {\text{TR}}_{\text{ARTbefore}}+{\text{ARTcurrent}}_{\text{t}}\times \\ & {\text{TR}}_{\text{ARTcurrent}} \end{array}$$

Where, BFTR_m,t_ = breastfeeding transmission rate at month; No_prophylaxis_m,t_ = HIV+ mothers with no prophylaxis/treatment; Prop_LT350_ = proportion of HIV+ mothers with no prophylaxis/treatment having CD4 counts < 350; TR _LT350_ = monthly transmission rate among HIV+ mothers with no prophylaxis/treatment having CD4 counts < 350; TR _GT350_ = monthly transmission rate among HIV+ mothers with no prophylaxis/treatment having CD4 counts ≥ 350; PropInc_t_ = HIV incidence women during breastfeeding; TR_i_ = tranmission rate for incident cases; OptA_m,t_ = HIV+ mothers using Option A in a month; TR_OptA_ = tranmission rate among HIV positive mothers who are on Option A; OptB_m,t_ = HIV+ mothers using Option B in a month; TR_OptB_ = tranmission rate among HIV positive mothers who are on Option B; ARTbefore_t_ = HIV+ mothers on-ART before current pregnancy; TR_ARTbefore_ = tranmission rate among HIV positive mothers who are on ART before current pregnancy; ARTcurrent_t_ = HIV+ mothers identified during current pregnancy; TR_ARTcurrent_ = tranmission rate among HIV positive mothers who are put on ART during current pregnancy.

The model estimates the number of new HIV infections among children in postnatal period using two factors: the number of exposed children (HIV-negative infants born to HIV+ mothers who are breastfeeding) and the monthly transmission rate during breastfeeding [20].

$${\text{CB}}_{\text{m},\text{t}}=\left(\text{BWt}–\Sigma_{\text{m}1=0\;\text{to}\;\text{m}\;-1}{\text{CB}}_{\text{m}1,\text{t}}\right)\times {\text{PropBF}}_{\text{m},\text{t}}\times \text{BFTRm},\text{t}$$

Where, CB_m,t_ = new infections occurring among children through breastfeeding in month ‘m’; BWt = the number of births occurring among WLHIV in year ‘t’; PropBFm,t = proportion of children born in year ‘t’ who are still breastfeeding at age ‘m’ months, and; BFTRm,t = breastfeeding transmission rate.

In the model, the children infected due to vertical tranmission would move into the subsequent age group based on age when they were infected and if they received any treatment [16, 22]. For the children estimated to have been infected perinatally but not put on ART, the median survival is less than two years. For the postnatal infections, the median longevity without ART ranges between 6 years to 14 years depending on the age at which infection occurs [23].

Among children living with HIV (CLHIV) who are getting the treatment, the annual AIDS-related mortality is informed by the International Epidemiological Databases to Evaluate AIDS (IeDEA) Consortium. CLHIV are also subjected to background mortality. When the CLHIV reaches the age-group of 5 years, they are distributed by CD4 category based on the IeDEA data. At age 15, CLHIV transition from the CD4 count categories associated with children 5–14 to the adult HIV states.

### MTCT-estimations related data inputs under the NACP of India

Periodic HIV burden estimations using Spectrum under the NACP of India is a practice since long [24–29]. Subnational Spectrum files, one file for each of the State/Union Territory (UT), are created for each round given the availability of the demographic, surveillance and programmatic data with epidemic starts year set at 1981 in each of the models. The inputted data includes ASFR since 1981 based on the Sample Registration System.

ART/prenatal prophylaxis coverage among pregnant WLHIV is inputted as zero before 2004 and then entered for single dose Nevirapine till 2012. For 2013, the coverage is entered for Option B (triple prophylaxis from 14 weeks). From 2014 onwards, the data is entered for Option B+ for ART started before and during the current pregnancy. Data entered on ART/prenatal prophylaxis coverage is specific to State/UT as reported in the NACP information system.

Retention on ART among pregnant WLHIV is kept as the default value of 85% for WLHIV on ART before the current pregnancy and at 80% for WLHIV put on ART during the current pregnancy across all State/UT-model. The data on ART/postnatal prophylaxis coverage for WLHIV identified during the breastfeeding period is not inputted given the lack of data on the same. Data on breastfeeding patterns among WLHIV is entered as same as that of HIV-negative women, specific for each State/UT, as informed by the rounds of the National Family Health Survey.

The model estimates vertical transmission separately for the peripartum (in utero and intrapartum) and postnatal (breastfeeding) period. The peripartum transmission probabilities, depending upon the on or off prenatal prophylaxis or ART regimen type, are applied to all deliveries among WLHIV to estimate the peripartum infections. The postnatal transmission probabilities, separate for the on or off prenatal prophylaxis or ART regimen type, are monthly probabilities applied to breastfeeding WLHIV during the whole of the breastfeeding period. Transmission due to incident infections during pregnancy or breastfeeding is modelled as a one-time risk. For all States/UTs models, default MTCT probabilities provided in the Spectrum model were used in HIV Estimations 2021 as in the previous rounds (see Table 1).

The Spectrum provides an exhaustive list of epidemiological indicators, adult as well as pediatric, including the number of people living with HIV (PLHIV), new HIV infections, AIDS-related deaths etc as an output of the modelling process. We present the analysis of vertical transmission by timing (prenatal or during breastfeeding) and by ART status (on or off prophylaxis or ART). The analysis includes the data for India and the high HIV burden States (either PLHIV size of ≥ 50,000 PLHIV or adult HIV prevalence of ≥1%) comprising Andhra Pradesh, Bihar, Delhi, Gujarat, Karnataka, Madhya Pradesh, Maharashtra, Manipur, Mizoram, Nagaland, Odisha, Punjab, Rajasthan, Tamil Nadu, Telangana, Uttar Pradesh and West Bengal. Table 2 summarizes the key characteristics of the States selected for the analysis [29, 30].

**Table 2:** Key epidemiological characteristics of the states selected.

State/UT	Adult (15–49 yrs) HIV prevalence (In %)	Total number of PLHIV (In thousand)	HIV incidence per 1000 uninfected population	Total number of annual new HIV infection (In thousand)	AIDS-related mortality per 100,000 population	Total number of AIDS-related deaths (In thousand)
Andhra Pradesh	0.67 (0.56–0.79)	321 (278–372)	0.08 (0.06–0.12)	4.39 (2.90–6.41)	17.40 (11.82–25.50)	9.19 (6.24–13.46)
Bihar	0.16 (0.11–0.22)	143 (96–197)	0.07 (0.03–0.13)	8.83 (3.95–15.70)	2.01 (0.86–4.00)	2.47 (1.06–4.93)
Delhi	0.31 (0.25–0.39)	56 (46–69)	0.14 (0.08–0.22)	2.74 (1.68–4.40)	4.61 (2.39–8.56)	0.95 (0.49–1.76)
Gujarat	0.19 (0.16–0.23)	114 (94–138)	0.04 (0.02–0.07)	2.51 (1.49–4.87)	1.16 (0.72–1.94)	0.81 (0.51–1.35)
Karnataka	0.46 (0.40–0.56)	276 (240–323)	0.06 (0.04–0.09)	3.79 (2.43–5.84)	10.09 (6.28–15.78)	6.74 (4.20–10.55)
Madhya Pradesh	0.08 (0.07–0.10)	55 (48–66)	0.02 (0.01–0.03)	1.54 (1.10–2.55)	1.18 (0.80–1.82)	1.00 (0.68–1.53)
Maharashtra	0.33 (0.28–0.39)	394 (341–457)	0.04 (0.03–0.07)	5.41 (3.42–8.06)	4.69 (3.20–7.48)	5.83 (3.99–9.31)
Manipur	1.05 (0.92–1.22)	28 (24–32)	0.32 (0.20–0.44)	0.98 (0.63–1.36)	26.59 (20.02–35.70)	0.84 (0.63–1.13)
Mizoram	2.70 (2.24–3.25)	24 (20–28)	1.31 (0.87–1.91)	1.55 (1.03–2.24)	15.80 (11.43–21.76)	0.19 (0.14–0.27)
Nagaland	1.36 (1.08–1.85)	22 (17–29)	0.51 (0.30–0.88)	1.10 (0.65–1.88)	13.98 (8.65–25.06)	0.31 (0.19–0.55)
Odisha	0.14 (0.10–0.19)	52 (39–69)	0.05 (0.02–0.08)	2.18 (1.02–3.77)	3.56 (1.72–5.95)	1.63 (0.79–2.72)
Punjab	0.28 (0.23–0.35)	73 (60–89)	0.05 (0.03–0.08)	1.38 (0.99–2.25)	1.91 (1.27–3.01)	0.58 (0.39–0.92)
Rajasthan	0.10 (0.09–0.12)	67 (56–80)	0.03 (0.02–0.05)	2.28 (1.27–3.68)	0.53 (0.36–0.79)	0.42 (0.29–0.63)
Tamil Nadu	0.22 (0.18–0.24)	163 (138–181)	0.02 (0.01–0.03)	1.50 (1.00–2.07)	2.72 (1.84–3.82)	2.08 (1.40–2.92)
Telangana	0.47 (0.37–0.60)	156 (129–194)	0.05 (0.03–0.11)	2.01 (0.99–4.03)	4.65 (2.98–8.42)	1.75 (1.12–3.18)
Uttar Pradesh	0.10 (0.08–0.14)	178 (139–236)	0.04 (0.02–0.07)	8.45 (4.46–15.15)	1.05 (0.58–1.96)	2.43 (1.35–4.53)
West Bengal	0.08 (0.07–0.09)	69 (63–78)	0.02 (0.01–0.02)	1.53 (1.06–2.27)	0.73 (0.55–1.02)	0.72 (0.54–1.00)
**India**	0.21 (0.17–0.25)	2401 (1992–2907)	0.05 (0.03–0.08)	62.97 (36.72–104.06)	3.08 (1.94–4.95)	41.97 (26.50–67.45)

## Results

Overall, around 5 thousand new HIV infections were estimated to happen as a result of vertical transmission during the peripartum (in utero and intrapartum) and the postnatal (breastfeeding) period in India in 2021 (Table 3). This included around 950 infections in Bihar, 600 each in Uttar Pradesh and Maharashtra, 400 in Andhra Pradesh, 325 in Karnataka, 200 each in Telangana and Odisha, 150 in Gujarat and Manipur each, 120 each in Delhi, Nagaland and Rajasthan and 100 in West Bengal. In the States of Madhya Pradesh, Mizoram, Tamil Nadu and Punjab, around 60–90 new infections were estimated to happen because of vertical transmission.

**Table 3:** MTCT rate in 2021, India and select states.

State/UT	PMTCT need	PMTCT Coverage (In %)	Vertical Transmissions (Number)	MTCT rate at 6 weeks (%)	Final Transmission Rate (%)
Andhra Pradesh	2,014 (1,700–2,421)	71.99 (59.24–85.29)	394 [261–576]	11.88 [8.95–14.86]	19.55 [15.35–23.81]
Bihar	2,446 (1,682–3,474)	23.14 (16.27–33.59)	956 [513–1517]	20.32 [15.73–22.80]	39.07 [30.71–44.15]
Delhi	394 (313–500)	52.03 (40.89–65.41)	119 [75–175]	15.65 [11.95–18.69]	30.17 [23.75–35.82]
Gujarat	1,030 (811–1,333)	90.47 (69.83–114.34)	152 [82–296]	7.48 [4.53–11.52]	14.80 [10.00–22.05]
Karnataka	1,707 (1,448–2079)	73.69 (60.17–86.85)	323 [221–478]	11.79 [9.14–14.69]	18.94 [15.10–23.14]
Maharashtra	536 (460–675)	60.27 (51.05–71.22)	600 [387–806]	13.52 [9.38–16.06]	22.52 [16.97–26.17]
Manipur	2,667 (2,252–3,145)	23.75 (20.22–28.73)	146 [113–181]	21.73 [20.09–23.38]	39.76 [36.49–42.48]
Madhya Pradesh	366 (303–430)	93.87 (74.47–109.34)	83 [59–151]	7.13 [5.53–11.35]	15.57 [12.67–22.56]
Mizoram	328 (271–399)	70.11 (57.58–84.83)	80 [50–115]	11.62 [8.11–14.57]	24.27 [18.29–29.45]
Nagaland	386 (300–538)	32.66 (23.36–41.69)	118 [78–184]	18.64 [15.54–21.22]	30.47 [25.70–34.79]
Odisha	534 (381–769)	43.07 (29.88–60.08)	189 [105–312]	17.71 [13.84–20.50]	35.32 [27.55–41.38]
Punjab	520 (411–670)	> = 95 (88.53–144.36)	61 [47–107]	5.67 [5.10–8.32]	11.80 [10.87–16.02]
Rajasthan	788 (624–961)	87.27 (71.17–109.74)	118 [63–195]	6.41 [3.98–9.63]	14.96 [9.94–20.68]
Tamil Nadu	774 (661–892)	> = 95 (92.01–124.39)	69 [46–102]	5.29 [3.69–7.36]	8.86 [6.78–11.73]
Telangana	1,073 (857–1,390)	75.74 (58.40–94.77)	195 [93–351]	10.23 [5.33–14.99]	18.21 [10.90–25.64]
Uttar Pradesh	2,186 (1,691–2,911)	50.78 (37.98–65.48)	610 [339–1015]	14.44 [9.43–18.36]	27.92 [19.62–34.86]
West Bengal	517 (460–599)	85.17 (73.41–95.60)	98 [72–135]	8.81 [6.81–11.22]	18.94 [15.31–23.04]
India	20612 [16379–26359]	63.88 [50.72–79.45]	4998 [3014–7811]	12.95 [9.45–16.02]	24.25 [18.50–29.50]

Nationally, the MTCT rate was at around 13% at 6 weeks in 2021. The estimated MTCT rate at 6 weeks was ~22% in Manipur, 20% in Bihar, ~18–19% in Nagaland and Odisha, ~13–16% in Delhi, Uttar Pradesh and Maharashtra and ~10–12% in Telangana, Mizoram, Karnataka, and Andhra Pradesh. In West Bengal, Gujarat and Madhya Pradesh, the estimated MTCT rate at 6 weeks ranged between >6% to <9% while in Punjab and Tamil Nadu, it ranged between >5% to <6%.

The final MTCT transmission rate, including the breastfeeding period, was estimated at around 24% nationally in 2021. The final transmission rate was more than 30% in Bihar, Delhi, Manipur, Nagaland, and Odisha. It was between 22–28% in Maharashtra, Mizoram and Uttar Pradesh and between >15–20% in Andhra Pradesh, Karnataka, West Bengal, Telangana and Madhya Pradesh. The final MTCT rate was between 10–15% in Rajasthan, Gujarat and Punjab and less than 10% in Tamil Nadu.

**Fig 1:**
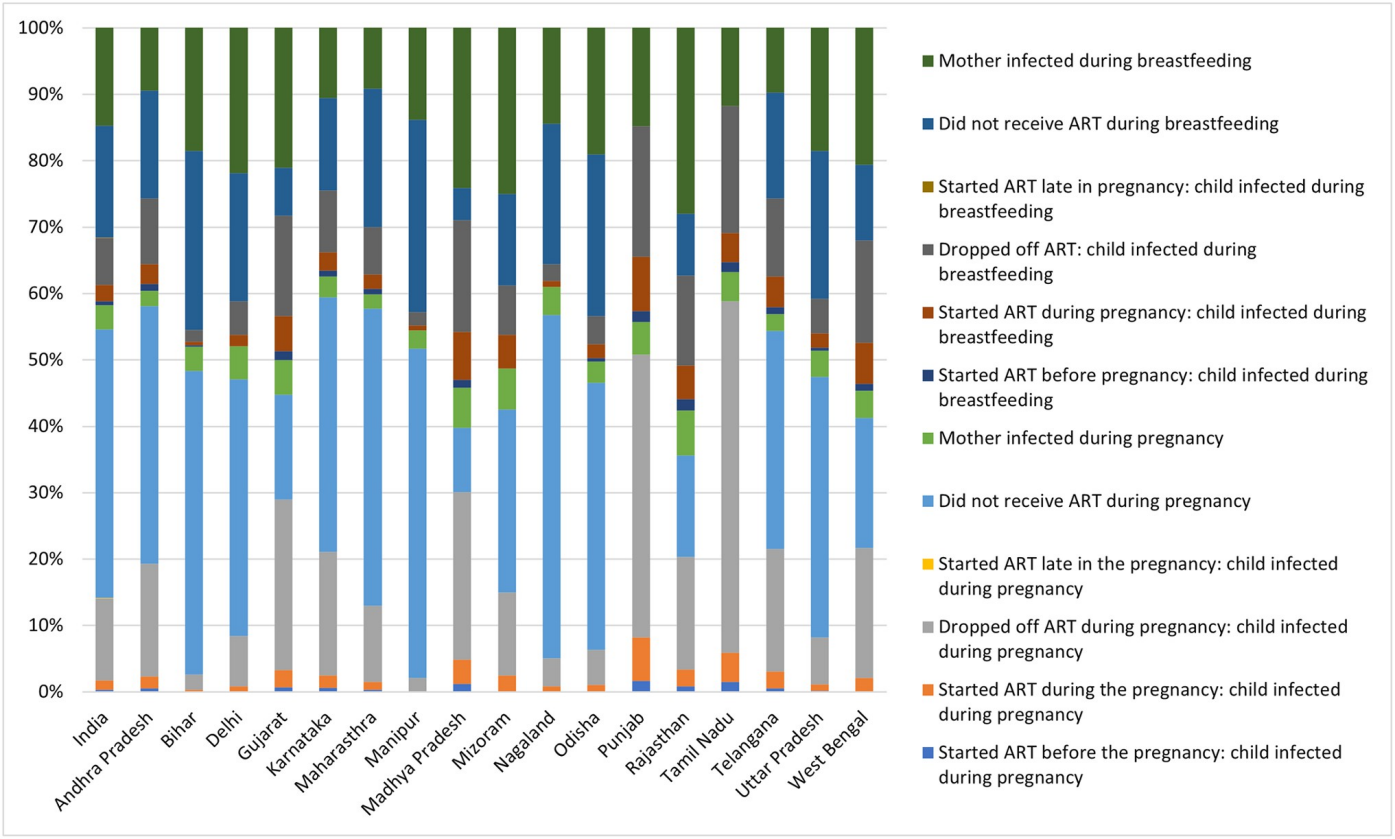
Stacked bar showing distribution of total estimated vertical transmission in 2021 by timing of infections and ART status.

**Fig 2:**
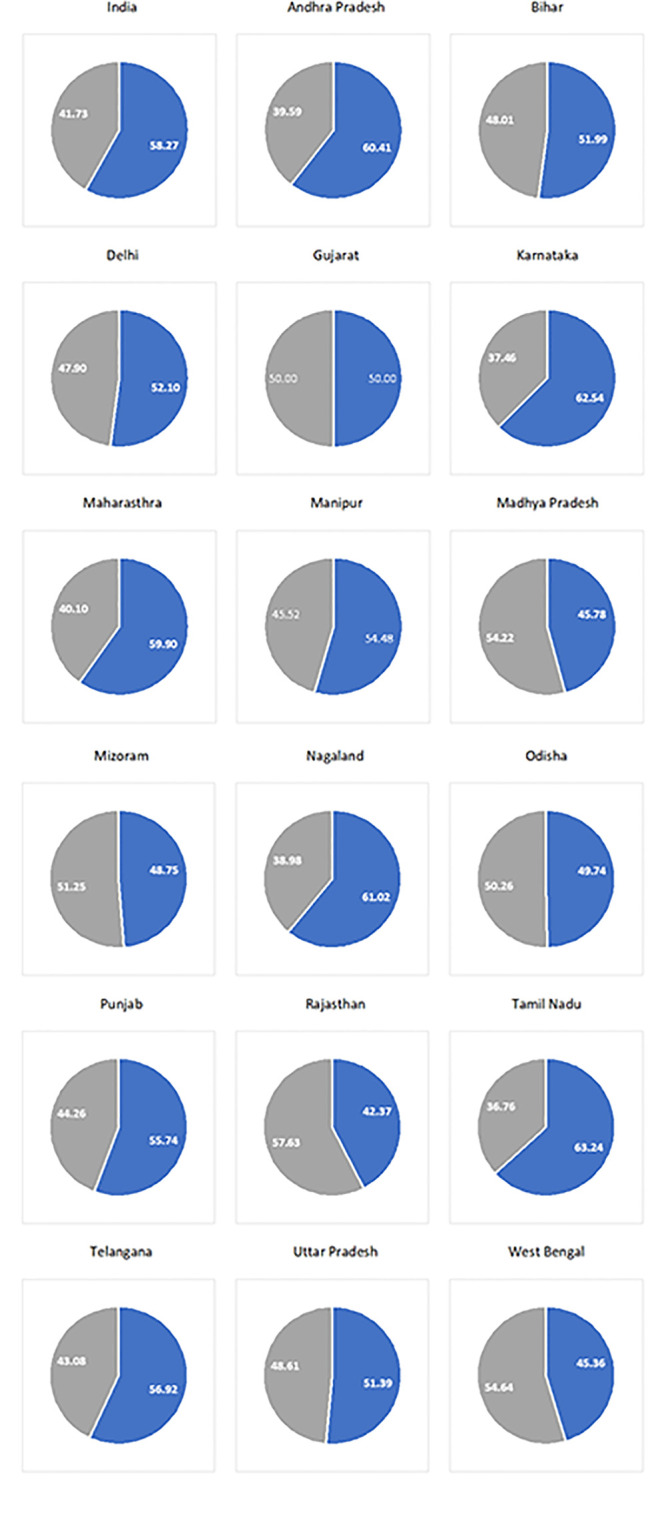
Proportional distribution of total estimated vertical transmission in 2021 by perinatal or post-natal infection.

Nationally, out of the total estimated 5,000 vertical transmissions in 2021, around 58% were estimated to happen during peripartum and rest during the breastfeeding period (Figs 1, 2 and Table 4). Overall, around two-fifth (40%) of total infections were peripartum infections among WLHIV who did not receive ART during pregnancy followed by around 12% among WLHIV who dropped off ART during pregnancy and another 4% among incident cases during the pregnancy. Around 17% of the total estimated vertical transmissions were attributed through breastfeeding among WLHIV who did not receive ART during breastfeeding followed by 15% among mothers who were incident cases during the breastfeeding period. Another 7% of the total estimated infections were postnatal infections among WLHIV who dropped off ART.

**Table 4:** Percentage distribution of total estimated vertical transmission in 2021 by the timing of infections and ART status.

State	Vertical Transmissions (Number)	Percentage distribution of total vertical transmission by the timing of infections and ART status									
		Perinatal Transmission					Postnatal Transmission				
		Started ART before the pregnancy	Started ART during the pregnancy	Dropped off ART during pregnancy	Did not receive ART during pregnancy	Mother infected during pregnancy	Started ART before the pregnancy	Started ART during the pregnancy	Dropped off ART	Did not receive ART during breastfeeding	Mother infected during breastfeeding
Andhra Pradesh	394 [261–576]	0.51	1.78	17.01	38.83	2.28	1.02	3.05	9.90	16.24	9.39
Bihar	956 [513–1517]	0.10	0.21	2.30	45.71	3.66	0.21	0.52	1.78	26.99	18.51
Delhi	119 [75–175]	0.00	0.84	7.56	38.66	5.04	0.00	1.68	5.04	19.33	21.85
Gujarat	152 [82–296]	0.66	2.63	25.66	15.79	5.26	1.32	5.26	15.13	7.24	21.05
Karnataka	323 [221–478]	0.62	1.86	18.58	38.39	3.10	0.93	2.79	9.29	13.93	10.53
Maharashtra	600 [387–806]	0.33	1.16	11.48	44.76	2.16	0.83	2.16	7.15	20.80	9.15
Manipur	146 [113–181]	0.00	0.00	2.07	49.66	2.76	0.00	0.69	2.07	28.97	13.79
Madhya Pradesh	83 [59–151]	1.20	3.61	25.30	9.64	6.02	1.20	7.23	16.87	4.82	24.10
Mizoram	80 [50–115]	0.00	2.50	12.50	27.50	6.25	0.00	5.00	7.50	13.75	25.00
Nagaland	118 [78–184]	0.00	0.85	4.24	51.69	4.24	0.00	0.85	2.54	21.19	14.41
Odisha	189 [105–312]	0.00	1.06	5.29	40.21	3.17	0.53	2.12	4.23	24.34	19.05
Punjab	61 [47–107]	1.64	6.56	42.62	0.00	4.92	1.64	8.20	19.67	0.00	14.75
Rajasthan	118 [63–195]	0.85	2.54	16.95	15.25	6.78	1.69	5.08	13.56	9.32	27.97
Tamil Nadu	69 [46–102]	1.47	4.41	52.94	0.00	4.41	1.47	4.41	19.12	0.00	11.76
Telangana	195 [93–351]	0.51	2.56	18.46	32.82	2.56	1.03	4.62	11.79	15.90	9.74
Uttar Pradesh	610 [339–1015]	0.16	0.98	7.04	39.28	3.93	0.49	2.13	5.24	22.26	18.49
West Bengal	98 [72–135]	0.00	2.06	19.59	19.59	4.12	1.03	6.19	15.46	11.34	20.62
India	4998 [3014–7811]	0.30	1.40	12.39	40.42	3.70	0.60	2.42	7.10	16.89	14.69

In Andhra Pradesh, sixty per cent of total estimated vertical transmissions in 2021 were transmission during the peripartum period (Figs 1, 2 and Table 4). Overall, around two-fifth (39%) of total infections were peripartum infections among WLHIV who did not receive ART during pregnancy followed by around 17% among WLHIV who dropped off ART during pregnancy and another 2% among babies whose mothers were incident cases during the pregnancy. Around 16% of the total estimated vertical transmissions were infections during the breastfeeding period among WLHIV who did not receive ART during breastfeeding followed by 10% among HIV-positive mothers who dropped off ART during breastfeeding. Another 9% of vertical transmission was among incident HIV infections during the breastfeeding period.

In Bihar, fifty-two per cent of total estimated vertical transmissions were attributed during the peripartum period. Overall, slightly less than half (46%) of total infections were peripartum infections among WLHIV who did not receive ART during pregnancy. Almost 27% of the total estimated vertical transmissions were infections during the breastfeeding period among WLHIV who did not receive ART during breastfeeding followed by another ~19% transmission during the breastfeeding period among mothers who were incident cases.

In Delhi, slightly more than half (52%) of total estimated vertical transmissions were transmission during the peripartum period. Overall, around 39% of total infections were peripartum infections among WLHIV who did not receive ART during pregnancy followed by around 19% during breastfeeding among WLHIV who did not receive ART during breastfeeding. Another ~22% transmission was during the breastfeeding period among mothers who were incident cases during breastfeeding and ~8% were during peripartum transmission among WLHIV who dropped off ART during pregnancy.

In Gujarat, almost 26% of the vertical transmission was peripartum transmission among WLHIV who dropped off ART during pregnancy followed by around 21% transmission during the breastfeeding period among mothers who were incident cases during breastfeeding. Around sixteen per cent of total infections were peripartum infections among WLHIV who did not receive ART during pregnancy. Another 15% of HIV-positive mothers who dropped off ART during breastfeeding.

In Punjab, slightly more than two-fifths (~43%) of the vertical transmission was peripartum transmission among WLHIV who dropped off ART during pregnancy. Another ~20% were transmission during the breastfeeding period among HIV-positive mothers who dropped off ART during breastfeeding. Around 15% of the estimated vertical transmission was during the breastfeeding period from mothers who were incident infections during breastfeeding.

In Tamil Nadu, more than half (53%) of the vertical transmission was peripartum transmission among WLHIV who dropped off ART during pregnancy. Another 19% were transmission during the breastfeeding period among HIV-positive mothers who dropped off ART during breastfeeding. Around 12% of the estimated vertical transmission was during the breastfeeding period from mothers who were incident infections during breastfeeding.

In Telangana, fifty-seven per cent of total estimated vertical transmissions happened during the peripartum period. Overall, almost one-third (33%) of total infections were peripartum infections among WLHIV who did not receive ART during pregnancy. Another 18% were estimated to be peripartum infections among WLHIV who dropped off ART during pregnancy. Around 16% of the postnatal infections were among WLHIV who did not receive ART during breastfeeding followed by 12% among those who dropped off ART during breastfeeding. Around 10% were postnatal infections among women who were incident cases.

In Uttar Pradesh, almost half (51%) of the total estimated vertical transmissions happened during the peripartum period. Overall, almost two-fifths (39%) of total infections were peripartum infections among WLHIV who did not receive ART during pregnancy while another 22% were postnatal infections among WLHIV who did not receive ART during breastfeeding. Another ~18% were postnatal infections among women who were incident cases.

In West Bengal, slightly less than half (46%) of the total estimated vertical transmissions happened during the peripartum period. Almost 40% of the total vertical transmission was perinatal transmission among WLHIV who were either not at all on ART during pregnancy or dropped off ART after initiating. Around 21% of the total estimated vertical transmissions were among incident cases during the breastfeeding period followed by another 15% among WLHIV who dropped off ART during breastfeeding.

## Discussions

The goal of eliminating mother-to-child transmission of HIV is integral to India’s commitment of achieving ending AIDS as a public health threat by 2030. With an overall national-level MTCT rate of 24% against the target of ≤ 5% being a breastfeeding population, it is evident that the country has to intensify the interventions, tailored to the local contexts, to accelerate the progress on the elimination of vertical transmission. This paper analyses the data from HIV estimations 2021 by States detailing the progress as well as the attributable causes for vertical transmissions.

Overall, around 95% of the total vertical infections in India are among three broad categories: 57% among HIV-positive mothers who did not receive ART during pregnancy or breastfeeding, 19% among mothers who dropped off ART during pregnancy or delivery, and 18% among mothers who were infected during pregnancy or breastfeeding. However, there are stark differences between States.

At least two-thirds of vertical transmission in Bihar, Maharashtra, Manipur, Nagaland and Odisha were among HIV-positive mothers who did not receive ART during pregnancy or breastfeeding. Andhra Pradesh, Delhi, Karnataka and Uttar Pradesh were other States where more than half of the vertical transmission were among HIV-positive mothers who did not receive ART followed by 40—<50% in Mizoram and Telangana.

Timely engagement in antenatal care (ANC), HIV testing and ART initiation are key intervention domains for responding to the missed opportunities of identifying HIV-positive mothers and subsequently initiating them on ART [31]. In some States like Nagaland and Bihar, only half of the mothers are having at least one ANC visit in the first trimester. In Uttar Pradesh, accounting for almost one-fourth of the total pregnant women, only around two-thirds had at least one ANC visit in the first trimester [30, 32]. Delayed registration in ANC care shortens the windows for offering complete packages of services including HIV testing and ART initiation.

Diagnosis of HIV during pregnancy/breastfeeding, the earlier the better, is fundamental to the initiation of the battery of services under EMTCT. HIV testing among pregnant women has seen rapid scale-up under the NACP with testing in 2021–22 almost three times of 2012–13. Incorporation of ‘opt out’ HIV testing services into routine ANC services and subsequent scale-up through decentralized models like facility-integrated model and community-based screening etc has driven this uptake. Still, even after not accounting for duplications, a total of 14 States/Union Territories in India, including Bihar, Delhi and Nagaland had less than 80 HIV tests for every 100 estimated pregnant women [30, 33]. EMTCT aims to test at least 95% of the estimated pregnant women for HIV as one of the three process indicators. Increasing the reach and uptake of HIV testing services in these 14 States would be critical to drive the elimination agenda under NACP.

Around 62–72% of total vertical transmissions in Tamil Nadu and Punjab is estimated among mothers who dropped off ART during pregnancy or breastfeeding followed by 30–41% in Gujarat, Madhya Pradesh, Rajasthan, Telangana and West Bengal. These are States with coverage of 75% or more against estimated EMTCT needs [30]. Improving the retention and adherence support will be critical elements for making progress in these States. A basket of community and facility-based interventions, tailored to the local context, has helped to improve retention and adherence during pregnancy and breastfeeding. Mentor mother approach, facility and community-based adherence support groups, community-level health providers empowered through information technology-enabled tools, and engagement of WLHIV’s social network including male partners have all worked in different settings [34–38].

Nationally, around one out of every five vertical transmissions (18%) is among mothers who were infected with HIV during pregnancy or breastfeeding and ranged from 11% in Maharashtra to around 35% in Rajasthan. Responding to the vertical transmission of HIV among mothers infected during pregnancy or breastfeeding would require strategies to identify such cases creating opportunities for diagnosing and initiating them on ART. In general, NACP recommends one test for pregnant women as a norm. Though WHO has recommended repeat HIV testing in high-incidence settings, studies in diverse settings, including in India, have demonstrated repeat HIV testing in the third trimester, during labour and/or during breastfeeding among women who tested HIV negative in the first test as a cost-effective strategy [39–41].

Our analysis provides an opportunity to understand the estimated number of vertical transmissions from the 2021 round of HIV burden estimations in India as a whole and by the major States, however, there are limitations which need to be taken into account to put the results in the context. Part of the limitations is attributed to the Spectrum model itself while part comes from limitations in the availability of local input data.

While Spectrum-based modelled estimates on the level and trends of the HIV/AIDS epidemic continue to be the workhorse of AIDS response globally, nationally and locally; limitations of model-based estimates are well documented [42–46]. The UNAIDS Reference Group on Estimates, Modelling and Projections regularly review the emerging science and provides technical guidance on the incorporation of the latest evidence into the model transmission parameters informing the periodic update on the HIV component of the Spectrum model [47, 48]. Still many assumptions on the age-sex patterns, fertility rates and mother-to-child-transmission probabilities, each with significant implications for the EMTCT estimates, are from settings which may have limitations in terms of representing country context [49]. International Epidemiology Databases to Evaluate AIDS (IeDEA) global consortium and Analysing Longitudinal Population-based HIV/AIDS data on Africa (ALPHA) network are two key sources periodically informing some of the fundamental assumptions under the Spectrum updates but with limited to no study sites in India [50–53].

Validation of modelled vertical transmission rate is another issue. In scenarios where it has been tested against real-world transmissions, it usually falls within the acceptable margin of error or closely meets observed rates [16, 54–56]. However, the validation has been done in only select settings as as there are very few studies, and those only in high-prevalence settings, describing the vertical tranmission rate at population level.

Limitations on EMTCT estimates also arise from sub-optimum alignment between strategic information being generated under the routine service delivery vis-à-vis model need. For example, the default value for retention to ART programmes of HIV-positive mothers at the time of delivery ranges from 80–85% [57]. This default value is used for each of the State/UT-model in India in the absence of evidence on this particular parameter under the programme. It is possible that at least in some of the high-performing States in the southern and western regions of the country, the retention of ART is higher than 85%. The assumption that the breastfeeding pattern among HIV-positive women is the same as that of HIV-negative women is another strategic information gap with potential implications on EMTCT estimates. Investments augmenting the available strategic information, through a complementing system of programme monitoring, surveillnace and research, for the generation of local evidence informing various parameters of HIV burden estimations would be vital to improving progress tracking on EMTCT under NACP Phase-V of India.

Despite the limitations, to the best of our knowledge, the analysis presented in this paper is the first of its kind to describe the MTCT rate by State/UTs. Understanding the vertical transmission by States, along with their attributable causes, is one of the most basic steps towards the attainment of EMTCT under NACP Phase-V. The two-pronged complementing strategy of improving the service delivery along with augmenting the strategic information would be a critical determinant. The study also provides opprtunity for further research to understand the inter-state variations. While the study describes the differences in MTCT and underlying causes between States, more can be done to give insight into the reasons for these differences augmenting the intervention focus.
